# Reaction kinetics of ultracold molecule-molecule collisions

**DOI:** 10.1038/s41467-018-07576-1

**Published:** 2018-12-07

**Authors:** Daniel K. Hoffmann, Thomas Paintner, Wolfgang Limmer, Dmitry S. Petrov, Johannes Hecker Denschlag

**Affiliations:** 1 Institut für Quantenmaterie and Center for Integrated Quantum Science and Technology (IQST), Universität Ulm, D-89069 Ulm, Germany; 2 LPTMS, CNRS, Univ. Paris Sud, Université Paris-Saclay, 91405 Orsay, France

## Abstract

Studying chemical reactions on a state-to-state level tests and improves our fundamental understanding of chemical processes. For such investigations it is convenient to make use of ultracold atomic and molecular reactants as they can be prepared in well defined internal and external quantum states. Here, we investigate a single-channel reaction of two Li_2_-Feshbach molecules where one of the molecules dissociates into two atoms 2AB ⇒ AB + A + B. The process is a prototype for a class of four-body collisions where two reactants produce three product particles. We measure the collisional dissociation rate constant of this process as a function of collision energy/temperature and scattering length. We confirm an Arrhenius-law dependence on the collision energy, an *a*^4^ power-law dependence on the scattering length *a* and determine a universal four body reaction constant.

## Abstract (web summary)

Chemical few-body reactions at ultralow temperatures exhibit scaling laws which are directly linked to the nature of the involved particles and their interactions. Here, the authors investigate the kinetics of four-body collision processes where diatomic molecules which are composed of ultracold fermionic atoms are either formed or dissociated.

## Introduction

The field of ultracold chemistry has been demonstrating an increasing level of control over internal and external states of atomic and molecular reactants^1–4^. However, even cold reactions have in general many possible final product states^5–15^ and reaction channels are therefore hard to track individually^16^. Nevertheless, reactions do exist where essentially only a single reaction channel is participating, such as atom-Feshbach molecule exchange reactions in Bose–Bose^17^ and Bose–Fermi^18^ mixtures, and three-body recombination A + A + B → AB + A in a Fermi–Fermi mixture^19^. Especially for reactions involving identical Fermions, the Pauli exclusion principle can lead to a particularly strong single-channel character and can also ensure a high collisional stability of AB molecules. By contrast, for a reaction A + B + D → AB + D, where A, B, and D are distinguishable atoms, the Pauli exclusion principle does not play a direct role. In the limit of zero-range interactions, the A–A–B and A–B–D systems belong to different universality classes, denoted non-Efimovian and Efimovian, respectively. Efimov physics and a broad range of associated phenomena have recently been studied to a great extent^20,21^. Here, we report on the first observation and characterization of the ultracold non-Efimovian reaction AB + AB → A + B + AB (break-up) and its inverse A + B + AB → AB + AB (three-body recombination). Besides being intrinsically four-body, these reactions also exhibit two- and three-body aspects. A peculiarity is that although the molecule AB is distinguishable from the atoms A and B, similar as for particle D, the large size of the weakly-bound AB molecule prevents, however, the system from being Efimovian. A simple dimensional analysis then suggests^22^ that at low energies the recombination rate constant reads *R*_2_ = *Cħa*^4^/*m*, where *a* is the AB scattering length, *m* is the atom mass, and *C* is a universal number.

In our experiments, we measure *R*_2_ and the break-up rate constant *C*_2_ using a gas of ^6^Li atoms in the lowest two hyperfine states denoted by A and B and weakly-bound AB dimers. By driving the mixture out of chemical equilibrium, we observe the subsequent reaction dynamics. Our measurements confirm the detailed-balance relation between these two constants^23^ *R*_2_ and *C*_2_ and, in particular, the Arrhenius law for the break-up reaction. We confirm the *a*^4^-dependence of *R*_2_ and provide the first experimental estimate for *C* ≈ 470. Our results are important for the stability problem of a pure atomic mixture, an issue proved to be relevant for the controversial topic of itinerant ferromagnetism (see, for example, refs. ^24,25^). Due to the different threshold laws and the large value of *C*, the reaction A + B + AB → AB + AB may be faster than A + A + B → AB + A, as soon as there is a sizeable seed of bound AB molecules in the system^23^.

## Results

### Experimental scheme

The initial atomic and molecular sample is prepared from an ultracold gas of *N*_tot_ = 2.6 × 10^5^ fermionic ^6^Li atoms, which consists of a balanced mixture of atoms in the two lowest hyperfine states $\left| {m_F = \pm 1{\mathrm{/}}2} \right\rangle$ of the electronic ground state. In the vicinity of the Feshbach resonance at *B*_0_ = 832.2 G (see ref. ^26^), exothermic three-body recombination can convert pairs of $\left| { - 1{\mathrm{/}}2} \right\rangle$, $\left| { + 1{\mathrm{/}}2} \right\rangle$ atoms into weakly-bound Feshbach molecules with the same well-defined internal quantum state. The process is reversible and a Feshbach molecule can dissociate again into the unbound $\left| { - 1{\mathrm{/}}2} \right\rangle$, $\left| { + 1{\mathrm{/}}2} \right\rangle$ atoms via an inelastic, endothermic collision with another molecule or atom. At thermal equilibrium balance of the back and forth reactions is established. This balance is a function of the particle densities, temperature, molecular binding energy, and scattering length, all of which can be controlled in our setup via confinement, evaporative cooling, and by choosing a magnetic offset field *B* < *B*_0_. Our trap is a combination of a magnetic trap and an optical dipole trap and is cigar shaped. The trap has a depth of *U*_0_ = 21 µK × *k*_B_, corresponding to a radial (axial) trapping frequency of *ω*_r_ = 2*π* × 0.99 kHz (*ω*_ax_ = 2*π* × 21 Hz), respectively. We use evaporative cooling to set the temperature to approximately 1.2–1.3 μK. At this temperature 80–90% of all atoms are bound in Feshbach molecules within the B-field range of 705–723 G of our experiments, corresponding to a binding energy *E*_b_ between 6 and 10 µK × *k*_B_ (see Methods). We note that at these settings where *T* ≥ *T*_F_ (*T*_F_ is the Fermi temperature) and *E*_b_ > *k*_B_*T*_F_, quantum degeneracy only plays a negligible role for the reaction kinetics.

**Fig. 1:**
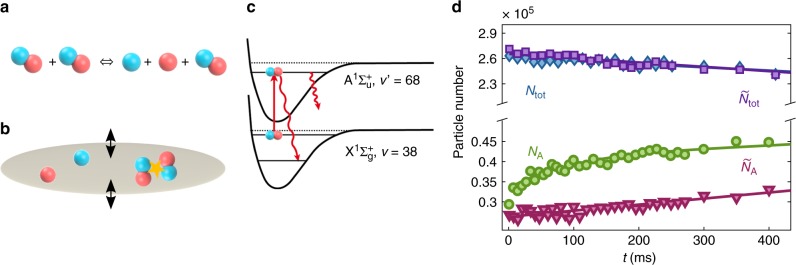
Molecule dissociation dynamics. **a** Detailed balance of collisional dissociation and association of dimers. **b** A parametric heating pulse triggers the reaction dynamics. **c** As part of the detection scheme, the Feshbach molecules which have a large admixture of the ${\mathrm{X}}^1{\mathrm{\Sigma }}_{\mathrm{g}}^ +$, *v* = 38 state are optically pumped to undetected atomic or molecular states via the intermediate level ${\mathrm{A}}^{\mathrm{1}}{\mathrm{\Sigma }}_{\mathrm{u}}^ +$, *v*′ = 68. **d** Measurement of dissociation dynamics at 709 G. Lower part: Circle (triangle) symbols show the number of unbound atoms *N*_A_ $\left( {\tilde N_{\mathrm{A}}} \right)$ for variable holding time *t* with (without) initial parametric heating pulse. Upper part: Diamond (square) symbols show the total particle number *N*_tot_ $\left( {\tilde N_{{\mathrm{tot}}}} \right)$ with (without) parametric heating pulse

In our first experiment, we suddenly raise the temperature of the gas using an excitation pulse of parametric heating. This shifts the gas out of thermal equilibrium and the gas responds by collisionally dissociating a part of its molecules, (see Fig. 1a). For this, we modulate the dipole trap depth (see Fig. 1b) with frequency *ω*_heat_ ≈ 1.7*ω*_r_ and amplitude ∆*U* = 0.21*U*_0_ during a period *t*_p_ = 20 ms. After the excitation, atoms and dimers thermalize on a time scale of a few milliseconds via elastic collisions, whereas the chemical equilibrium requires a much longer time of 150 ms.

### Dynamics

To investigate these dynamics, we measure how the number of molecules *N*_M_ and the number of unbound atoms *N*_A_ change as a function of time. We measure *N*_A_ by using standard absorption imaging. However, prior to the imaging we first remove all Feshbach molecules from the gas. For this, a resonant laser pulse transfers the molecules to an electronically excited molecular state ${\mathrm{A}}^{\mathrm{1}}{\mathrm{\Sigma }}_{\mathrm{u}}^ +$, *v*′ = 68 which subsequently decays within a few ns to undetected atomic or molecular states^27,28^ (see Fig. 1c), see Methods. The laser pulse has a duration of 0.5 ms, which is short compared to the reaction dynamics. In order to determine *N*_M_, we measure in a second run the total number of atoms *N*_tot_ = 2*N*_M_ + *N*_A_, whether they are bound or unbound, and subtract *N*_A_. For this we use again absorption imaging. The Feshbach molecules are so weakly bound that the imaging laser resonantly dissociates them quickly into two cold atoms which are subsequently detected via absorption imaging^29^.

Figure 1d shows the measurements of *N*_A_ and *N*_tot_ as a function of holding time after the heating pulse. While the total number of atoms *N*_tot_ is essentially constant apart from some slow background losses, the atom number *N*_A_ exhibits a 30% increase in about 100 ms which is the dissociation response of Li_2_ molecules to the thermal pulse. Besides this, *N*_A_ also exhibits a slow, steady increase which we attribute to a background heating of the gas, e.g., due to spontaneous photon scattering of the dipole trap light (see Supplementary Note 1). As shown by $\tilde N_{\mathrm{A}}$ in Fig. 1d, this background heating is also present in the absence of the initial heating pulse. Similarly, the slow decay of *N*_tot_ is also present without the heating pulse (see $\tilde N_{{\mathrm{tot}}}$ in Fig. 1d). It can be completely explained by inelastic collisions between molecules as previously investigated in ref. ^30^.

In principle, collisional dissociation in our experiment can be driven either by atom–molecule collisions or by molecule–molecule collisions. We only consider molecule–molecule dissociation since its rate is about two orders of magnitude larger in our experiments than for atom–molecule dissociation with its known rate constant of^23^ *C*_1_ ≈ 10^−13^ cm^3^ s^−1^ and given the fact that the mean density of atoms is a factor of ten smaller than for the dimers. In a simple physical picture, the suppression of the atom–dimer dissociation is due to the Pauli principle acting on the outgoing channel, which involves two identical fermionic atoms^31,32^. In the molecule–molecule collisional dissociation, the molecules can either dissociate into four unbound atoms, 2AB ⇒ 2A + 2B, or into two unbound atoms, 2AB ⇒ AB + A + B. However, since in our experiments the molecular binding energy *E*_*b*_ is typically by a factor of 5 larger than the thermal energy *k*_B_*T*, the dissociation into four atoms comes at an additional sizeable energy cost and is therefore comparatively suppressed by an Arrhenius factor of exp(−*E*_b_*/k*_B_*T*) ≈ 7 × 10^−3^, see also ref. ^23^. Therefore, to first order, we only need to consider dissociation into two atoms. The evolution of the density *n*_A_ of unbound atoms is then given by the rate equation,

1 $$\dot n_{\mathrm{A}} = 2C_2n_{\mathrm{M}}^2 - R_2n_{\mathrm{A}}^2n_{\mathrm{M}}{\mathrm{/2}}$$

Here, *n*_M_ is the molecule density and *C*_2_ (*R*_2_) are the rate constants of molecule dissociation (association). A spatial integration of Eq. (1) gives the rate equation for the number of unbound atoms,

2 $$\dot n_{\mathrm{A}} = \left( {4\pi ^{3{\mathrm{/}}2}} \right)^{ - 1}\frac{{C_2}}{{\sigma _{\mathrm{r}}^2\sigma _{{\mathrm{ax}}}}}N_{\mathrm{M}}^2 - 2^{ - 7}\left( {2\pi ^2} \right)^{ - 3{\mathrm{/}}2}\frac{{R_2}}{{\sigma _{\mathrm{r}}^4\sigma _{{\mathrm{ax}}}^2}}N_{\mathrm{A}}^2N_{\mathrm{M}}$$

where we assume a Boltzmann distribution in a harmonic trap. Here, $\sigma _{{\mathrm{r}}\left( {{\mathrm{ax}}} \right)} = \sqrt {k_{\mathrm{B}}T{\mathrm{/}}2m\omega _{{\mathrm{r}}\left( {{\mathrm{ax}}} \right)}^2}$ denote the radial (axial) cloud width of the molecular gas and *m* is the mass of ^6^Li. Furthermore, in Eq. (2) we have used the fact that the cloud size for the unbound atoms is $\sigma _{{\mathrm{r}}\left( {{\mathrm{ax}}} \right),{\mathrm{A}}} = \sqrt 2 \sigma _{{\mathrm{r}}\left( {{\mathrm{ax}}} \right)}$. By fitting Eq. (2) to the data of Fig. 1d we can determine the rate coefficients to be *C*_2_ = (2.0 ± 0.6) × 10^−12^ cm^3^ s^−1^ and *R*_2_ = (4.1 ± 1.2) × 10^−22^ cm^6^ s^−1^. For the fit we use the measured widths *σ*_r(ax)_, which turn out to be fairly constant during the holding time *t* (a more detailed discussion will be given below).

### Temperature dependence

**Fig. 2:**
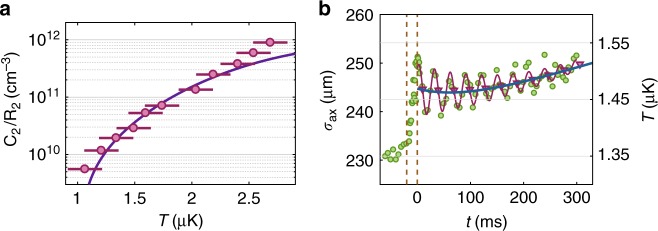
Temperature dependence of the equilibrium state and temperature evolution. **a** The ratio *C*_2_/*R*_2_ (circles) is plotted as a function of temperature *T* at *B* = 723 G. The error bars denote the s.d. in the thermometry. The continuous line is a calculation without any free parameters (for details see section "Temperature dependence"). **b** Measured evolution of the axial cloud size *σ*_ax_ (green circles) at *B* = 705 G after injecting a heat pulse during −20 ms < *t* *<* 0 ms (vertical dashed lines). The heat pulse abruptly increases the temperature *T* and size *σ*_ax_ ∝ $\sqrt T$. In addition, it excites small collective breathing mode oscillations, see the red line as a guide to the eye. The red triangles mark the evolution of *σ*_ax_ when averaged over one oscillation period. This evolution is well described by a model calculation (blue line) as described in Supplementary Note 1 and Supplementary Fig. 1. The temperature scale applies to the non-oscillatory part of the data

Next, we investigate how the reaction rates depend on temperature. For this, it is convenient to study the atom molecule system in a state of near equilibrium, where $\dot N_{\mathrm{A}}$ ≈ 0, i.e., $\dot N_{\mathrm{A}}$ is much smaller than the individual collisional dissociation/association rates of Eq. (2). We can then set ${\textstyle{{C_2} / {R_2}}} = 2^{ - 5}\left( {2\pi } \right)^{ - 3{\mathrm{/}}2}{\textstyle{{\textstyle{{N_{\mathrm{A}}^2} / {N_{\mathrm{M}}}} {\sigma _{\mathrm{r}}^2\sigma _{{\mathrm{ax}}}}}}}$. Thus, a change in the ratio *C*_2_/*R*_2_ due to a variation in temperature can be experimentally observed in terms of a change of particles numbers and widths. Figure 2a shows such measurements at *B* = 723 G ($\dot N_{\mathrm{A}}$ was always at least a factor of ten smaller than the collisional dissociation/association rates). Within the small temperature range between 1 and 3 µK, the rate constant ratio ${\textstyle{{C_2} / {R_2}}}$ increases by more than two orders of magnitude. This result can be compared to a prediction based on statistical mechanics^23^,

3 $${\textstyle{{C_2} / {R_2}}} = h^{ - 3}\left( {\pi mk_{\mathrm{B}}T} \right)^{3{\mathrm{/}}2}{\mathrm{e}}^{ - E_{\mathrm{b}}{\mathrm{/}}k_{\mathrm{B}}T}$$

which is shown in Fig. 2a as a continuous line with no adjustable parameters. The agreement between experiment and theory is quite good. The strong increase of ${\textstyle{{C_2} / {R_2}}}$ with temperature is dominated by the Arrhenius law exponential ${\mathrm{e}}^{ - E_{\mathrm{b}}{\mathrm{/}}k_{\mathrm{B}}T}$, which comes into play for the endothermic dissociation (*C*_2_) but is absent for the exothermic recombination process (*R*_2_).

The strong temperature dependence of the rate constants potentially has a strong influence on the reaction dynamics of our atom/molecule system, as the chemical reactions change the temperature of the gas. To quantify this influence, we take a closer look at the temperature evolution in our experiment by tracking the cloud size *σ*_ax_ ∝ $\sqrt T$, see Fig. 2b. Initially the system is in thermal equilibrium and the molecular cloud has an axial size of about *σ*_ax_ = 230 μm, which corresponds to a temperature of *T* ≈ 1.3 μK (see Supplementary Note 2 and Supplementary Fig. 2). The heating pulse, which starts at *t* = −20 ms and ends at *t* = 0, deposits thermal energy in the system. Due to the fast elastic collisions of dimers and atoms the thermal energy deposition results in a fast increase of the cloud size of about 6% which corresponds to a temperature increase of ∆*T* ≈ 0.15 μK. In addition, the modulation of the dipole trap during the heating pulse excites unwanted breathing mode oscillations in the cloud with a small amplitude of 2%. The mean cloud size which is obtained by averaging over one oscillation (red circles in Fig. 2b) is almost constant within the first 150 ms after the heating pulse. This might be at first surprising since one might expect the endothermic dissociation to considerably lower the temperature again. However, since the initial atom number is quite small, only a small amount of molecules need to break up to significantly increase the recombination rate $\propto N_{\mathrm{A}}^2$ and thus to reach a new balance. Therefore only a small amount of the injected heat is consumed for the dissociation, corresponding to a small amount of cooling. Moreover, this residual cooling is almost canceled by the background heating. As a consequence, the remaining temperature variation is less than 1%. For later times, *t* > 150 ms, when the reaction triggered by the heating pulse has already stopped, the background heating leads to a monotonically increasing mean cloud size. From our results in Fig. 2a we conclude that a temperature variation of 1% leads to *C*_2_*/R*_2_ variations of at most a few percent, which is negligible with respect to our current measurement accuracy.

In view of these complex dynamics, we have set up a system of coupled differential equations that describe in a more complete fashion the various reaction/loss processes at varying temperatures (see Supplementary Note 1). The solid curve in Fig. 2b is a result of these calculations which in general show very good agreement with our measurements.

### Interaction strength dependence

**Fig. 3:**
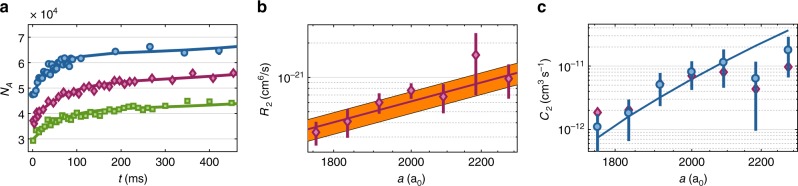
Dependence of the reaction rate constants on the scattering length. **a** Reaction dynamics for three scattering lengths of *a* = (1760, 1920, 2000)*a*_0_ (green squares, red diamonds, and blue circles), corresponding to the magnetic fields *B* = (705, 711, 714) G, respectively. The continuous lines are fits based on Eq. (2) from which *R*_2_ and *C*_2_ can be extracted. **b** The measured association rate constant *R*_2_ as a function of *a* (red diamonds). *R*_2_ roughly follows the universal relation *R*_2_ = *Cħa*^4^/*m*, with *C* = 470 obtained from a fit to the data (red continuous line). The majority of the data can be found in a band (orange area) around the fit curve. The band is bounded by 370 < *C* < 570. **c** The dissociation rate constant *C*_2_ (red diamonds) as a function of *a*. The temperatures between the individual measurements varied by about 15%. To compensate the influence of the temperature, we use Eq. (4) to rescale *C*_2_ to values corresponding to *T* = 1.5 µK (blue circles). The error bars correspond to temperature uncertainties and the 95% confidence bounds determined by fits as in **a**. The blue continuous line is the theoretical prediction of Eq. (4) for a universal constant of *C* = 470

Finally, we investigate the influence of the interaction strength between the particles on the reaction dynamics. For this, we tune the scattering lengths with the help of the magnetic B-field. We note that the dimer–dimer scattering length *a*_dd_ is given by *a*_dd_ = 0.6*a*, where *a* is the scattering length for atom–atom collisions^33^. Figure 3a shows three measurements for *a* = (1760, 1920, 2000)*a*_0_. For technical reasons, we start with three different *N*_A_ at *t* = 0. However, this has negligible influence on the dynamics of the dissociation, which we have checked with a numerical calculation. Already from the data shown in Fig. 3a it is obvious that the dissociation rates strongly increase with the scattering length. From fits to these and additional measurements we extract *R*_2_(*a*) and *C*_2_(*a*) for various scattering lengths and plot the results on a double logarithmic scale in Fig. 3b, c (red diamonds).

The association (three-body recombination) process characterized by the rate constant *R*_2_ has been extensively discussed for various Efimovian systems^34–36^, where it depends on the three-body parameter, and for non-Efimovian Fermi–Fermi mixtures, where it is suppressed in the low-energy limit^31,32^. By contrast, here we are dealing with a non-Efimovian system of three distinguishable particles, for which a simple dimensional analysis^22^ predicts the low-energy threshold law *R*_2_ = *Cħa*^4^/*m*, where *C* is a universal constant. In Fig. 3b, this *a*^4^ scaling behavior is plotted for *C* = 470, obtained from a fit to our data. Our results show quite good agreement with the expected power law dependence within the error bars. Figure 3c shows *C*_2_ for various *a* (red diamonds). These data are still raw in the sense that each measurement is taken at a slightly different temperature which increases with the scattering length (see Supplementary Note 3). In order to compensate this temperature change, we use Eq. (3) to rescale the measured *C*_2_ rate constants to values corresponding to a constant temperature *T* = 1.5 µK (see blue circles in Fig. 3b). The resulting rate constant *C*_2_ increases by more than one order of magnitude in the tuning range and agrees reasonably with the theoretical prediction (without any free parameter),

4 $$C_2 = C\frac{{\left( {\pi mk_{\mathrm{B}}} \right)^{1{\mathrm{/}}2}k_{\mathrm{B}}}}{{2h^2}}T^{3{\mathrm{/}}2}a^4e^{ - E_{\mathrm{b}}\left( a \right)/{k_{\mathrm{B}}T}},$$

which is obtained by inserting *R*_2_ = *Cħa*^4^/*m* into Eq. (3) and using again *C* = 470. As far as we know there is no direct theoretical prediction for this number. D’Incao and co-workers^37^ calculated dimer–dimer elastic and inelastic scattering properties in a wide range of collision energies. For the energy interval relevant here, these calculations indicate 30 ≲ *C* ≲ 100 which is also consistent with our own numerical estimates based on refs. ^33,38^. The large discrepancy between the theoretical and experimental value needs to be investigated in future studies. It may be due to an atom dimer attraction in the *p*-wave channel (see supplemental material of ref. ^39^), which is difficult to take into account theoretically within our current approach.

## Discussion

In conclusion, we have investigated the collisional dissociation of ultracold molecules in a single reaction channel which is characterized by the precisely defined quantum states of the involved atoms and molecules. Using a heating pulse we shift an atom/molecule mixture which is initially in detailed balance out of equilibrium and measure the evolution of the system until it reaches a new equilibrium. This allows us to determine reaction rate constants, in particular for the collisional dissociation of two molecules. Furthermore, we find a strong temperature dependence of this rate which is consistent with the well known Arrhenius equation. In addition, we find agreement of the association (dissociation) rate constant with a scaling behavior of $a^4\left( {a^4e^{ - E_{\mathrm{b}}{\mathrm{/}}k_{\mathrm{B}}T}} \right)$, respectively. From our data we estimate the universal constant *C* ≈ 470, which is in discrepancy with the theoretical prediction. For the future, we plan to extend the current work to study the dynamics of chemical reactions in a regime, where Fermi and Bose statistics play an important role.

## Methods

### Preparation of the atomic and molecular quantum gas

To prepare our sample of ultracold atoms and molecules, we initially trap 10^9 6^Li atoms in a magneto-optical trap, where the atoms are cooled to a temperature of 700 µK. The particles are transferred to an optical dipole trap of a focused 1070 nm laser beam with an efficiency of 1%. To generate a balanced distribution (50%/50%) of atoms in the $\left| {m_F = \pm 1{\mathrm{/}}2} \right\rangle$ spin states we apply a resonant 100 ms radio frequency pulse. Initially the optical trap has a depth of 4 mK × *k*_B_ and is subsequently ramped down within 6 s to 1.3 µK × *k*_B_ to perform forced evaporative cooling. This is carried out at a magnetic field of 780 G and during this process Feshbach molecules form via three-body recombination. To suppress particle loss in the experiments and to assure harmonicity of the trapping potential, the trap depth is ramped up again to *U*_0_ = 21 µK × *k*_B_ after evaporation. We then ramp the B-field in a linear and adiabatic fashion to the specific value at which the experiment will be carried out, within the range of 705–723 G. After a holding time of 100 ms, the gas has a temperature of approximately 1.2–1.3 µK and is in chemical equilibrium, with 80–90% of all atoms being bound in Feshbach molecules. The binding energy of the molecules can be determined from^40^ $E_{\mathrm{b}} = {\textstyle{{\hbar ^2} / {m\left( {a - \bar a} \right)^2}}}\left( {1 + 2.92{\textstyle{{\bar a} /({a - \bar a}}}) - 0.95{\textstyle{{\bar a^2} / {\left( {a - \bar a} \right)^2}}}} \right)$ using $\bar a$ = 29.9*a*_0_, which yields values between 6 and 10 µK × *k*_B_ in our B-field range. The scattering length *a* as a function of the B-field is taken from ref. ^26^. It can be approximated with $a = a_{{\mathrm{bg}}}\left( {1 - {\textstyle{{{\mathrm{\Delta }}B} / ({B - B_0}}}}) \right)$, where ∆*B* = −263.3 G is the width of the resonance and *a*_bg_ = −1582 *a*_0_ is the background scattering length.

### Removing of Feshbach molecules

To optically pump the Feshbach molecules into undetected states, we use a 673 nm laser with a peak intensity of *I*_0_ = 500 mW cm^−2^ which excites all Feshbach molecules to the ${\mathrm{A}}^1{\mathrm{\Sigma }}_{\mathrm{u}}^ +$, *v*′ = 68 state^27^ (see Fig. 1c) within 500 µs. The excited molecular state decays within a few ns either into two unbound atoms which quickly leave the trap or into deeply bound Li_2_ molecules which are invisible for our detection.

Besides molecule excitation, the pulse leads to photoassociation of unbound atoms. This reduces the number of free atoms and leads to an overestimation of the molecule number. However, in our parameter range and for our free atom densities the resulting error for the free atom number is below 1% and can be neglected (see also ref. ^28^).

## Electronic supplementary material

Supplementary Information

## Data Availability

The datasets generated and/or analyzed during the current study are available from the corresponding author on reasonable request.
