# Supplementary material for: Reaction kinetics of ultracold molecule-molecule collisions
Source: Nat Commun. 2018 Dec 7;9:5244. doi: 10.1038/s41467-018-07576-1 (PMC6286306; doi:10.1038/s41467-018-07576-1)
Supplement: Supplementary file 1 — Supplementary Information [file 41467_2018_7576_MOESM1_ESM.pdf]

REACTION KINETICS OF ULTRACOLD MOLECULE-MOLECULE COLLISIONS

Hoffmann et al.

SUPPLEMENTARY INFORMATION

Supplementary Note 1: Modelling of the reaction dynamics

We perform model calculations to describe in more detail the measured dynamics of the atom/ molecule system, triggered by the initial heat pulse. For this, we integrate the following coupled system of rate equations for the atom number N_A, the molecule number N_M and the temperature T,

$$\dot{N}_\mathrm{A} = C_2\, a_1 \frac{N_\mathrm{M}^2}{\sigma_\mathrm{r}^2\, \sigma_\mathrm{ax}} - R_2\, a_2 \frac{N_\mathrm{M}\, N_\mathrm{A}^2}{\sigma_\mathrm{r}^4 \sigma_\mathrm{ax}^2} \tag{1}$$

$$\dot{N}_\mathrm{M} = -\frac{\dot{N}_\mathrm{A}}{2} - C_\mathrm{DD}\, a_1 \frac{N_\mathrm{M}^2}{2\, \sigma_\mathrm{r}^2\, \sigma_\mathrm{ax}} \tag{2}$$

$$\dot{T} = -\frac{E_\mathrm{b}}{6 k_\mathrm{B}\, (N_\mathrm{A} + N_\mathrm{M})} \dot{N}_\mathrm{A} + C_\mathrm{H} \tag{3}$$

where $a_1 = (4\pi^{3/2})^{-1}$ and $a_2 = 2^{-7}(2\pi^2)^{-3/2}$ are numerical constants. Equation (1) is identical to eq. (2) in the main text. The first term in eq. (2) corresponds to the conversion between molecules and unbound atoms, while the second term accounts for molecule losses in inelastic dimer-dimer collisions with rate constant of $C_\mathrm{DD} = 2.3 \times 10^{-13}\,\mathrm{cm}^3\mathrm{s}^{-1}$, which is extracted from the previous measurement of ref.[1]. Equation (3) has two contributions. The first one accounts for cooling due to endothermic dissociation and heating due to exothermic recombination reactions. The second contribution corresponds to background heating of the gas caused, e.g., by off-resonant scattering of the dipole-trap light. Equations (1) and (2) are coupled via the cloud sizes $\sigma_\mathrm{r(ax)} = \sqrt{k_\mathrm{B} T / m_\mathrm{M} \omega_\mathrm{r(ax)}^2}$ to the temperature equation (3).

The results of a corresponding calculation at 709 G are shown in Supplementary Fig. 1 and are in very good agreement with the experimental data. The values of the parameters C_2 and R_2 are the same as in the main text. $C_\mathrm{H} = (3.0 \pm 1.0) \times 10^{-7}\,\mathrm{s}^{-1}$ is mainly determined by the long-time evolution of the cloud size (see Supplementary Fig. 1d,e).

The total particle number in Supplementary Fig. 1c) exhibits the losses caused by the inelastic dimer-dimer collisions and agrees well with the experimental measurements. The temperature in Supplementary Fig. 1d) first decreases slightly due to endothermic dissociation after the heat pulse and then increases again due to the dipole-laser induced photodissociation. Supplementary fig. 1e shows the calculated cloud width σ_ax as determined by the temperature T. It agrees well with the measurements (green circles) if we average over the small-amplitude collective oscillations which have been excited by the initial heat pulse.

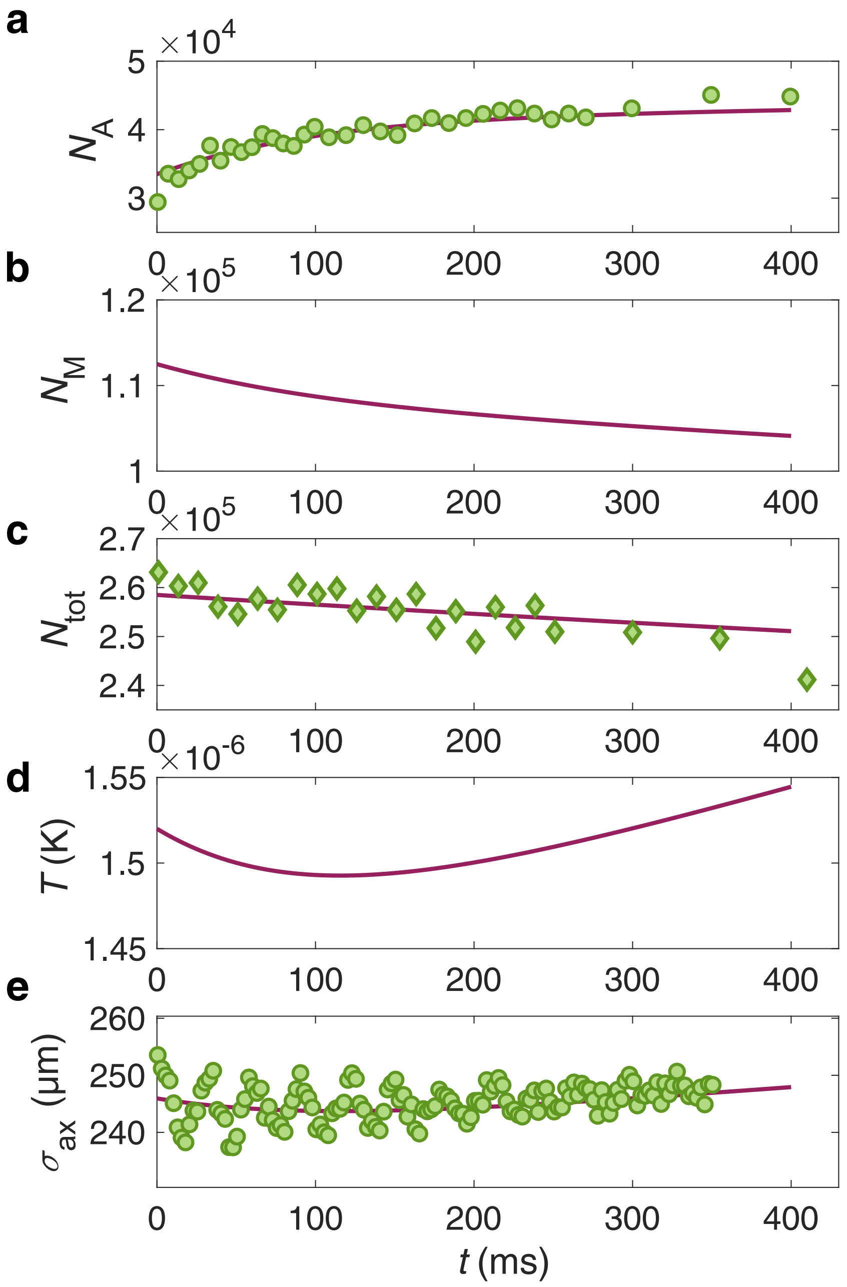

Supplementary Figure 1. Model calculation for the evolution of the atom/ molecule system. Results from the coupled differential equations (1-3) (continuous lines). Plot symbols show experimental data for 709 G. For details see text of Supplementary Note 1.

Supplementary Note 2: Thermometry

Within the parameter range of our experiments the molecular and atomic density distributions are each well described by that of a non-interacting thermal gas located in a harmonic trap. The

axial sizes of the molecular and atomic clouds are given by $\sigma_{\mathrm{ax}} = \sqrt{k_{\mathrm{B}}T/m\omega_{\mathrm{ax}}^2}$ and $\sigma_{\mathrm{ax,A}} = \sqrt{2}\sigma_{\mathrm{ax}}$, respectively. Because the axial trapping frequency ω_{ax} is precisely known for our setup, we can determine the temperature T by measuring the molecular or atomic cloud size. These cloud sizes are extracted from images of the mixed atom/ molecule clouds after careful analysis, for which we also determine the atom fraction $N_{\mathrm{A}}/N_{\mathrm{tot}}$ of the cloud. Supplementary Figure 2 shows a calculated typical example for the 1D density distributions (where the transverse directions have been integrated out) for atoms n_{A}, molecules n_{M} and both $n_{\mathrm{tot}} = 2n_{\mathrm{M}} + n_{\mathrm{A}}$.

Supplementary Note 3: Rescaling the measured C_2 to a constant temperature

Supplementary table 1 is the list of temperatures at which the C_2 measurements in fig. 3c are taken. The statistical uncertainty of the temperatures is around $\Delta T = 0.08\,\mu\mathrm{K}$.

The temperatures increase with decreasing scattering length a. This temperature change is a result of the way we prepare the sample. In particular, the magnetic field ramp to the target field takes place within a $21\,\mu\mathrm{K}\times k_{\mathrm{B}}$ deep trap which prevents further evaporative cooling. With

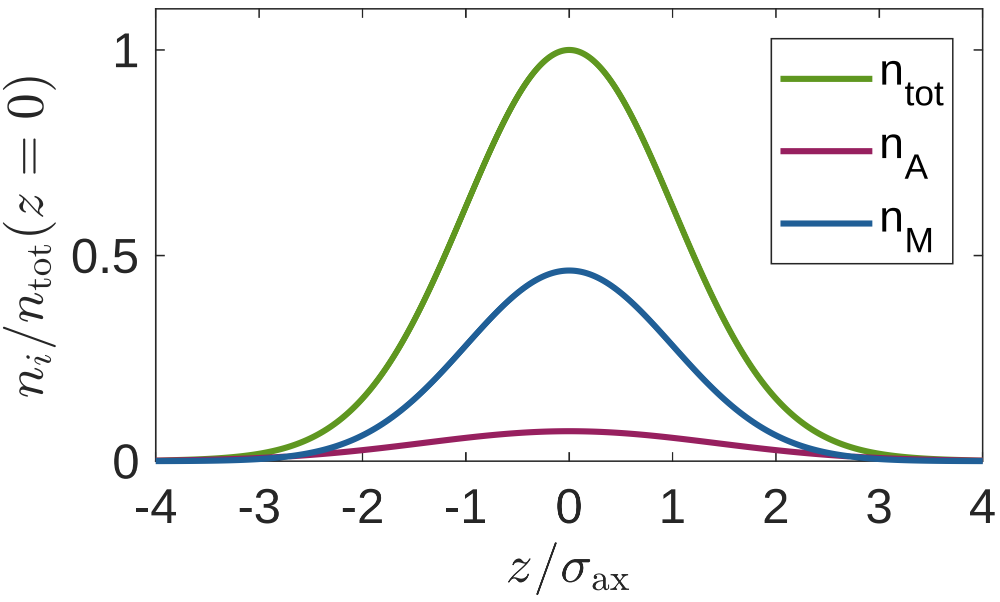

Supplementary Figure 2. Calculated 1D density distribution for a thermal cloud of atoms and molecules. In a harmonic potential non-interacting atoms and molecules exhibit a Gaussian density distributions n_{A} and n_{M}, respectively, of which the widths differ by a factor or $\sqrt{2}$. The total density distribution is the sum $n_{\mathrm{tot}} = 2n_{\mathrm{M}} + n_{\mathrm{A}}$. Here, the atom fraction $N_{\mathrm{A}}/N_{\mathrm{tot}}$ is 0.6.

Supplementary Table 1. Temperatures and scattering lengths of the measurements in fig. 3c.

a (a_0)	1760	1830	1920	2000	2090	2180	2280
T (μK)	1.60	1.52	1.51	1.47	1.42	1.41	1.35

decreasing a, the binding energy of the dimers increases and therefore for a given temperature the equilibrium molecule fraction increases. The corresponding molecular association, however, heats the sample.

SUPPLEMENTARY REFERENCES

[1] Bourdel, T. et al. Experimental Study of the BEC-BCS Crossover Region in Lithium 6. *Phys. Rev. Lett.* **93**, 050401 (2004).
